# Cost of care and antibiotic prescribing attitudes for community-acquired complicated intra-abdominal infections in Italy: a retrospective study

**DOI:** 10.1186/1749-7922-9-39

**Published:** 2014-06-20

**Authors:** Lidia Dalfino, Francesco Bruno, Sergio Colizza, Ercole Concia, Andrea Novelli, Fabrizio Rebecchi, Federico Spandonaro, Cristina Alato

**Affiliations:** 1 Anesthesia and Intensive Care Unit - Emergency and Organ Transplantation Department, University of Bari, Policlinico of Bari, P.zza G. Cesare 11, 70124 Bari, Italy; 2 Department of General Surgery, Fatebenefratelli-Isola Tiberina, Rome, Italy; 3 Clinical Infectious Disease, Department of Pathology, University of Verona, Verona, Italy; 4 Department of Health Sciences, Clinical Pharmacology and Oncology Section, University of Florence, Florence, Italy; 5 Digestive, Colorectal, Oncologic and Minimally Invasive Surgery, Department of Surgical Sciences, University of Turin, Turin, Italy; 6 CEIS, University of Rome Tor Vergata, Rome, Italy

**Keywords:** Antibiotics, Community-acquired intra-abdominal infections, Cost of care, Direct costs, Hospitalization

## Abstract

**Introduction:**

Complicated intra-abdominal infections (cIAIs) are a common cause of morbidity worldwide, and in spite of improvements in patient care, therapeutic failure still occurs, impacting in-hospital resource consumption. This study aimed to assess the costs associated with the treatment of community-acquired cIAIs, from the Italian National Health Service perspective.

**Methods:**

This retrospective study analyzed the charts of patients who were discharged from four Italian university hospitals between January 1 and December 31, 2009 with a primary diagnosis of community-acquired cIAIs. Patient characteristics, diagnosis, surgical procedure, antibiotic therapy, and length of hospital stay were all recorded and the cost of total hospital care was estimated. Costs were calculated in Euros at 2009 values.

**Results:**

The records of 260 patients (mean age 48.9 years; 57% males) were analyzed. The average cost of care for a patient hospitalized due to cIAI was €4385 (95% CI 3650–5120), with an average daily cost of €419 (95% CI 378–440). Antibiotic therapy represented just under half (44.3%) of hospitalization costs. The strongest predictor of the increase in hospital costs was clinical failure: patients who clinically failed received an average of 8.2 additional days of antibiotic therapy and spent 11 more days in hospital compared with patients who responded to first-line therapy (both p < 0.05 vs. patients who were successfully treated). Furthermore, they incurred €5592 in additional hospitalization costs (2.88 times the cost associated with clinical success) with 53% (€2973) of the additional costs attributable to antibiotic therapy. Overall, antibiotic appropriateness rate was 78.8% (n = 205), and was significantly higher in patients receiving combination therapy compared with those treated with monotherapy (97.3% vs. 64.6%).

**Conclusion:**

The results of this study suggest that hospitals need to be aware of the clinical and economic consequences of antibiotic therapy of cIAIs and to reduce overall resource use and costs by improving the rate of success with appropriate initial empiric therapy.

## Introduction

Intra-abdominal infections (IAIs), encompassing a wide spectrum of pathological conditions from uncomplicated appendicitis to fecal peritonitis, are a common cause of morbidity worldwide. IAIs are defined as complicated (cIAIs) when infection extends beyond the affected hollow viscus into the peritoneal space, causing either localized or diffuse peritonitis [1]. In spite of improvements in patient care, therapeutic failure still occurs in patients with community-acquired (CA) cIAIs [2-5], highly impacting in-hospital resource consumption [2,5,6]. In early European series, patients with community-acquired cIAIs who clinically failed had significantly longer length of hospital stay and incurred significantly higher inpatient charges than those who were treated successfully [2,6]. More recently, the economic rebound of clinical failure has been investigated in a large US multi-institutional database of 6056 patients with cIAIs, showing an additional 4.6 days spent in hospital and inpatient charges of $6368 when clinical failure occurred [5].

The clinical outcome of patients with cIAIs is heavily influenced by prompt surgical source control and timely effective antimicrobial treatment [7,8]. Current guidelines recommend a wide range of first-line single or multiple antimicrobial regimens based on patient characteristics (comorbidities, immunosuppression, and previous antibiotic exposure), expected involved pathogens (inferred by source and origin, community or hospital-acquired, of infection) and local resistance epidemiology [1,5]*.*

Most recent guidelines also consider the antibiotic treatment of cIAIs from a microbiological point of view, particularly in terms of pathogens producing ESBLs (Extended Spectrum Beta-Lactamases). For community-acquired extrabiliary cIAIs, empirical antimicrobial therapy can be divided into categories: treatment for critically ill and non-critically ill patients, and treatment for both groups according to the presence or absence of risk factors for ESBL-producing pathogens. In non-critically ill patients, amoxicillin-clavulanate or ciprofloxacin-metronidazole are possible options, but in the presence of risk factors for ESBL these are not sufficient, and other drugs such as tigecycline and ertapenem are useful. In critically ill patients without risk factors for ESBL, piperacillin-tazobactam is an option, but in the presence of ESBL risk factors carbapenems like imipenem and meropenem are more appropriate [9].

Of note, knowledge of antibiotic drugs costs is suggested as additional criteria supporting clinical decision-making [1,5,9]. In fact, in some US and European studies, a significant influence of empiric antibiotic therapy choice on economic outcome of cIAIs has emerged [3,6,7,10]. However, the wide inter-country variability of antimicrobial prescribing attitudes and of health care and reimbursement systems organization could differently impact on cost estimates. Therefore, due to this limited generalizability of data, referring to pharmacoeconomic analyses from other countries could be misleading.

To the best of our knowledge, a costs analysis of cIAIs hospital care has never been performed in Italy, although IAIs have been ranked as the second most common infectious reason for hospitalization, after respiratory infections [11]. To address this issue, this study aimed to assess the costs associated with the treatment of community-acquired cIAIs, from the Italian National Health Service (i.e. the third payer) perspective.

## Methods

### Study design

This one-year, multicentre, retrospective, incidence-based observational study was performed in four Italian (Bari, Florence, Turin, and Verona) acute-care university hospitals. The study was conducted in accordance with the ethical principles of the Declaration of Helsinki (and subsequent revisions) and to the current norm for observational studies. The protocol was reviewed and approved by each study site’s ethical committees. Due to the retrospective study design, informed consent was not deemed necessary.

### Patient selection

Patients were identified by searching computerized hospitalization records of each recruiting hospital for a primary discharge diagnoses of one of five International Classification of Diseases 9th revision (ICD-9) codes suggestive of cIAIs (540.1- acute appendicitis with intra-abdominal abscess, 540.0 - acute appendicitis with diffuse peritonitis, 567.2 - other suppurative peritonitis, 567.8- other specified peritonitis, 567.9 - unspecified peritonitis, 567.0 - peritonitis in infectious disease classified elsewhere. Patients were eligible for inclusion if they (1) were hospitalized between January 1 and December 31, 2009; (2) were at least 18 years old at the time of their hospitalization; (3) had a primary discharge diagnosis suggesting any cIAIs; (4) underwent laparotomy, laparoscopy or percutaneous drainage of an intra-abdominal abscess and (5) received intravenous antibiotics.

### Patient analysis

A review of each patient’s chart was performed, and relevant parameters were recorded in standardized individual electronic case report forms. These included: patient age, gender, comorbidities (diabetes mellitus, obesity or others), patient lifestyle factors (smoking, alcoholism), known risk factors for antibiotic failure [1,9] (cancer, liver cirrhosis, acute liver failure, renal failure, end stage renal failure, anemia, leukopenia, coagulopathy, immunosuppression, or others), primary and secondary discharge diagnoses, primary surgical procedure and unplanned additional surgeries (if any), laboratory, instrumental and microbiology tests (number, type and results), antibiotic therapy type, dose, and duration, switch to second-line antibiotic drugs and reasons for the switch (clinical failure, antibiotic resistance, adverse event, unspecified), illness severity markers (use of artificial nutrition, antifungal drugs, immune globulins, central venous catheter, renal replacement therapies, mechanical ventilation), medical specialists’ consultancies (type and frequency), length of hospital stay, and discharge status (alive/dead). Hospital ward of admission, in-hospital transfers (to other wards or to the intensive care unit [ICU]), and place of discharge (home, other hospitals or long-term care facilities) were also recorded.

### Definitions

*Primary surgical procedures* were categorized according to the source of infection as surgical operations on upper gastrointestinal (GI) tract (biliary or gastro-duodenal tract, and small intestine), gall-bladder, appendix, lower GI tract (colon-rectum), peritoneal abscesses drainage, or others.

*Clinical success* was defined as patient recovery with either first line empiric antibiotic therapy or a step-down from initial therapy (transition wide/narrow spectrum or intravenous/oral).

*Clinical failure* was defined as a switch to second-line antibiotic treatment, need for unscheduled additional abdominal surgeries, or patient death [2-4,6,7].

*First-line empiric antibiotic therapy* was defined as a regimen started at the time of surgical intervention, before the availability of any culture data.

*Switch to second-line antibiotic therapy* was defined as the addition of one or more parenteral antibiotics to the initial antibiotic regimen or as a complete or partial switch of the initial antibiotic regimen to another parenteral antibiotic regimen.

*Unscheduled additional abdominal surgeries* were taken into account if they occurred 2 or more days after the primary surgical procedure and were related to poor primary source control. Secondary procedures were not considered in the analysis when there was a mention of other reasons (i.e. technical issues or hemorrhage) that might have led to re-operation.

First-line empiric antibiotic therapy was defined as *appropriate* if all isolated bacteria were sensitive to at least one of the antibiotics administered in patients with documented positive intra-abdominal swabs or blood cultures. Alternatively, in patients with negative or no cultures, empiric therapy was deemed as appropriate when the selected regimen covered enteric gram-negative aerobic and anaerobic bacteria and drug dosing was adequate, according to current guidelines [1]. Antibiotic regimens not fulfilling the above criteria were defined as *inappropriate*.

*Leucocytosis* was defined by a white blood cell (WBC) count >12,000/mm^3^. *Leukopenia* was defined as a WBC count <4000/mm^3^.

### Cost analysis

A estimate of the cost of antibiotics was performed by multiplying the number of antibiotic days by the unit price of that antibiotic and by the number of per day doses. The overall cost of antibiotic treatment for each patient was the sum of costs calculated for all parenteral antibiotics received by the patient during the hospitalization period. The unit price of antibiotics was based on official ex-factory prices per unit in Italy [12]. Laboratory tests, instrumental tests, and specialists’ consultancies utilization were directly recorded and their costs were assessed by referring to fees for providers of specialist services recognized by the Italian National Health Service (I-NHS). Costs related to primary surgical procedures were not included in analysis, as we assume they were independent of the adopted antibiotic therapy.

Other direct costs, including personnel, ordinary maintenance and hotel costs, were indirectly estimated by using Diagnosis-Related Group’s tariffs per admission provided to hospitals by the I-NHS. Specifically, this estimate was based on the acknowledged over-threshold per hospital day tariff, which is the per day cost to hospitals for length of stay prolonged over an *a priori* defined threshold (i.e. a tariff applicable to length of stay statistically considered as outliers), assuming that by subtracting the average costs of specialist services provided from this tariff, an acceptable proxy of the general cost sustained for patient management could be obtained.

Costs were expressed in Euro values at the time they were incurred (year 2009 values).

### Statistical analysis

Sample size derived from the number of cIAIs patients meeting inclusion criteria, admitted at the four recruiting centers from January 1 to December 31, 2009.

Continuous data are expressed as means ± standard deviation or 95% confidence intervals (CIs), and categorical data as number of events and percentages. Univariate statistical analysis was performed by student t-test or chi-squared test, as appropriate, to compare baseline characteristics and outcomes of clinical success and failure groups.

Due to the retrospective design of the study, a regression model by means of a backward stepwise model selection approach was employed to investigate the independent hospital charges predictors, in order to control for confounding factors and obtain the exact contribution of each parameter to the outcome variable. The model takes into account patient status and controls for type of primary surgical procedure, unplanned additional surgeries, and antibiotic therapy switches. Considered variables were dummy. In order to avoid co-linearity between variables, a Pearson correlation was performed. Covariates in the model were: patient age and gender, one or more high risk factors, primary surgical procedure, surgical approach, antibiotic monotherapy/combination therapy, clinical success/failure, one or more therapeutic failure risk factors, unplanned additional surgeries, more than one additional surgery.

Statistical analyses were performed by using SPSS statistical software version 15.1 (SPSS Inc., Chicago, IL, USA). A *P* value <0.05 was considered statistically significant.

## Results

### Patient characteristics

**Figure 1:**
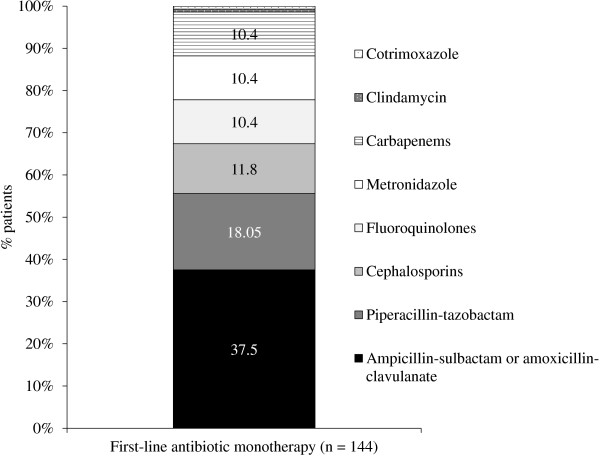
**Antibiotics administered to patients who received monotherapy for first-line treatment of complicated intra-abdominal infections.** Cephalosporins included: cefazolin, ceftizoxime, cefotaxime, and ceftriaxone; fluoroquinolones included: ciprofloxacin and levofloxacin; carbapenems included imipenem and meropenem; aminoglycosides included: amikacin, gentamicin and tobramycin.

**Figure 2:**
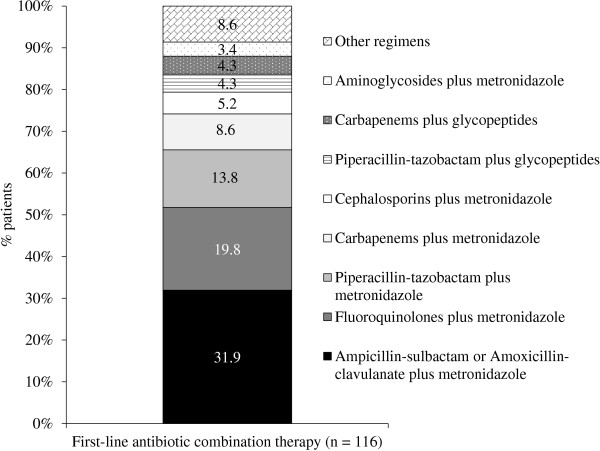
**Antibiotic regimens administered to patients who received combination therapy for the first-line treatment of complicated intra-abdominal infections.** Cephalosporins included: cefazolin, ceftizoxime, cefotaxime, and ceftriaxone; fluoroquinolones included: ciprofloxacin and levofloxacin; carbapenems included imipenem and meropenem; aminoglycosides included: amikacin, gentamicin and tobramycin. Other regimens included: aminoglycosides plus ampicillin/sulbactam or piperacillin/tazobactam, or imipenem (n = 4), fluoroquinolones plus amoxicillin/clavulanate, cephalosporins, tygecicline or piperacillin/tazobactam (n = 5), fluoroquinolones plus clindamycin (n = 1).

A total of 260 patients (mean age 48.9 years; 57% males) met the study entrance criteria. On hospital arrival, 250 (96.2%) patients were admitted to surgical wards, 8 (3.1%) to medical wards, and 2 (0.7%) to the ICU. The majority of patients (62.3%) were affected by complicated appendicitis. Patients were surgically approached by laparoscopy in slightly more than half of cases, and by laparotomy in the majority of the others (Table 1).One-hundred forty-four (55.4%) patients received first-line empiric antibiotic therapy as a monotherapy drug regimen, with the most frequent being ampicillin-sulbactam or amoxicillin-clavulanate (37.5%), and piperacillin-tazobactam (18.05%; Figure 1). In the remaining 116 (44.6%) patients, who received combination antibiotic therapy, the most common treatments were amoxicillin-clavulanate or ampicillin-sulbactam (31.9%), fluoroquinolones (19.8%), or piperacillin-tazobactam (13.8%), all in combination with metronidazole (Figure 2).

**Table 1:** Demographic and clinical characteristics

**Characteristic**	**Patients (n = 260)**
Mean ± SD age, years	48.9 ± 20
Males, n (%)	149 (57.3)
Comorbidities, n (%)	
Diabetes mellitus	12 (4.6)
Obesity	12 (4.6)
Lifestyle factors, n (%)	
Smoking	27 (10.4)
Alcoholism	0 (0)
Therapeutic failure risk factors, n (%)	
Age > 65 years	63 (24.2)
Cancer	16 (6.2)
Anemia	16 (6.2)
Liver cirrhosis	1 (0.4)
Renal failure	1 (0.4)
Acute liver failure	0 (0)
End stage renal failure	2 (0.8)
Coagulopathy	2 (0.8)
Immunosuppression	2 (0.8)
Leukopenia	0 (0)
Primary surgical intervention site, n (%)	
Appendix	162 (62.3)
Lower GI tract	51 (19.6)
Upper GI tract	13 (5.0)
Gall-bladder	14 (5.4)
Peritoneal abscess	16 (6.1)
Explorative laparotomy/laparoscopy	4 (1.5)
Surgical approach, n (%)	
Laparoscopy	135 (51.9)
Laparotomy	116 (44.6)
Percutaneous	9 (3.5)
Illness severity markers, n (%)	
Parenteral nutrition	52 (20.0)
Central venous catheter	44 (16.9)
Antifungal drugs	28 (10.8)
Enteral nutrition	22 (8.4)
Invasive mechanical ventilation	20 (7.7)
Immune globulins	0 (0)
Renal replacement therapies	0 (0)
ICU transfer, n (%)	24 (9.2)
Mean ± SD length of hospital stay, days	10.4 ± 13
Mortality rate, n (%)	6 (2.3)

GI, gastrointestinal; ICU, intensive care unit; SD, standard deviation.

Of the 48 microbiologically evaluable patients (18.4% of the total patient population), 23 (47.9%) intra-operative abdominal site cultures (21 peritoneal swabs, and 2 intra-operative biopsies), 12 (25.0%) abdominal drainage fluid cultures, 11 (22.9%) blood cultures and 2 (4.2%) surgical wound swabs were performed. Among 34 (70.8%) documented positive cultures, the most frequent isolated pathogen was *Escherichia coli* (58.8%), followed by *Klebsiella pneumoniae* (14.7%).

Due to the low representation of the microbiological evaluable population, antibiotic therapy appropriateness was inferred by covered antimicrobial spectrum and dosing adequacy of starting empiric regimens, as detailed in the methods section. Overall, antibiotic appropriateness rate was 78.8% (n = 205), and was significantly higher in patients receiving combination therapy compared with those treated with monotherapy (97.3% vs. 64.6%). Clinical success chances with appropriate antibiotic therapy were 78.5% (n = 161) and 34.5% (n = 19) with inappropriate therapy.

In total, 194 (74.6%) patients responded to antibiotic treatment and experienced clinical success (clinical success group), while 66 (25.4%) patients did not respond to antibiotic therapy (clinical failure group). Ninety-six per cent (95.8%) of patients were discharged to home, 1.5% to long-term care facilities, 0.4% to another hospital, and 2.3% died in hospital.

### In-hospital charges

The average cost of care for a patient hospitalized due to cIAI was €4385 (95% CI 3650–5120), with an average daily cost of €419 (95% CI 378–440). Antibiotic therapy cost by itself represented just under half (44.3%) of hospitalization costs. Clinical failure was the strongest independent predictor of hospitalization costs increases in multivariable regression analysis, followed by unscheduled additional abdominal surgeries, combination antibiotic therapy administration, patient comorbidities and illness severity markers (R^2^ = 0.47) (Table 2).

**Table 2:** Independent predictors of hospitalization costs associated with complicated intra-abdominal infection

	**Not standardized coefficients**		**Standardized coefficients**	**t**	***P *value**	**Cost variation (%)**
	**B**	**Standard error**	**Beta**			
Constant	3,733.00	793.44		4.705	0.000	
Clinical failure	3,817.85	681.02	0.275	5.606	0.000	+87.04
Unscheduled secondary surgeries	4,558.00	1,059.75	0.226	4.301	0.000	+104
Antibiotic combination therapy	2,264.09	580.05	0.186	3.903	0.000	+51.6
Comorbidities	2,177.45	742.28	0.14	2.933	0.004	+49.6
Therapeutic failure risk factors	1,755.84	675.91	0.137	2.598	0.010	+40
Appendectomy	−3,481.79	698.81	−0.279	−4.982	0.000	−79.4
Cholecystectomy	−2,920.24	1,339.50	−0.109	−2.180	0.030	−66.6
Female gender	−1,043.09	572.92	−0.085	−1.821	0.070	−23.8

The critical influence of clinical outcome on hospitalization costs prompted us to investigate clinical characteristics and economic outcome of patients stratified into clinical failure and success groups (Table 3). Compared with the clinical success group, patients in the clinical failure group were older and were more likely to have cancer. More patients in the clinical failure group had undergone lower GI tract surgical procedures, were surgically approached by laparotomy, and had markers indicative of severe disease and required ICU transfer (Table 3). Moreover, they more frequently received antibiotic monotherapy (69.7% vs. 52.1%). Specifically, patients who failed therapy were more like to have received metronidazole monotherapy (21.4% vs. 3.03%) and were less likely to have received the combination of fluoroquinolones plus metronidazole (4.7% vs. 22.6%) as their first-line antibiotic therapy.

**Table 3:** Demographic and clinical characteristics of patients stratified by clinical outcome

**Characteristic**	**Clinical success group (n = 194)**	**Clinical failure group (n = 66)**	***P *value**
Mean ± SD age, years	46.4 ± 19	56.2 ± 21	<0.05
Males, n (%)	113 (58.2)	36 (54.5)	NS
Comorbidities, n (%)			
Diabetes mellitus	7 (3.6)	5 (7.5)	NS
Obesity	9 (4.6)	3 (4.5)	NS
Lifestyle factors, n (%)			
Smoking	22 (11.3)	5 (7.5)	NS
Alcoholism	0 (0)	0 (0)	NS
Therapeutic failure risk factors, n (%)			
Age > 65 years	38 (19.5)	25 (37.8)	<0.05
Cancer	8 (4.1)	8 (12.1)	<0.05
Anemia	6 (3.1)	10 (15.2)	<0.05
Liver cirrhosis	1 (0.5)	0 (0)	NS
Renal failure	1 (0.5)	1 (1.5)	NS
End stage renal failure	2 (1.0)	0 (0)	NS
Coagulopathy	2 (1.0)	0 (0)	NS
Immunosuppression	1 (0.5)	1 (1.5)	NS
Primary surgical intervention site, n (%)			
Appendix	132 (68.0)	30 (45.4)	<0.05
Lower GI tract	23 (11.8)	28 (42.4)	<0.05
Upper GI tract	10 (5.1)	3 (4.5)	NS
Gall-bladder	13 (6.7)	1 (1.5)	NS
Peritoneal abscess	13 (6.7)	3 (4.5)	NS
Other	3 (1.5)	1 (1.5)	NS
Surgical approach, n (%)			
Laparoscopy	111 (57.2)	24 (36.3)	<0.05
Laparotomy	76 (39.2)	40 (60.6)	<0.05
Percutaneous	7 (3.6)	2 (3.0)	NS
Antibiotic treatment, n (%)			
Monotherapy	101 (52.1)	46 (69.7)	<0.05
Combination therapy	93 (47.9)	20 (30.3)	<0.05
Illness severity markers, n (%)			
Parenteral nutrition	27 (13.9)	25 (37.8)	<0.05
Central venous catheter	16 (8.2)	24 (36.3)	<0.05
Antifungal drugs	12 (6.2)	16 (24.2)	<0.05
Enteral nutrition	10 (5.2)	12 (18.2)	<0.05
Invasive mechanical ventilation	6 (3.1)	14 (21.2)	<0.05
ICU admission, n (%)	6 (3.1)	18 (27.3)	<0.05
Mortality rate, n (%)	0 (0)	6 (9.1)	NS

GI, gastrointestinal; ICU, intensive care unit; NS, not significant; SD, standard deviation.

The majority of patients who experienced clinical failure (99.6%) switched to second-line antibiotic therapy, 12 (18.2%) underwent unscheduled additional surgeries and 6 (9.1%) died. Second-line antibiotic therapy included switching to entirely different antibiotics in 63.6% of cases and addition of one or more drugs to the initial antibiotic regimen in 36.3% of cases. Reasons for switching therapy were clinical ineffectiveness in 63.6% of patients, microbiologic resistance in 9% and was unreported in 24.2% of patients. Second-line regimens involved meropenem (25.7%), ertapenem (21.2%), tygecicline (19.6%) and glycopeptides (10.6%).

### In-hospital charges by therapeutic outcome

**Figure 3:**
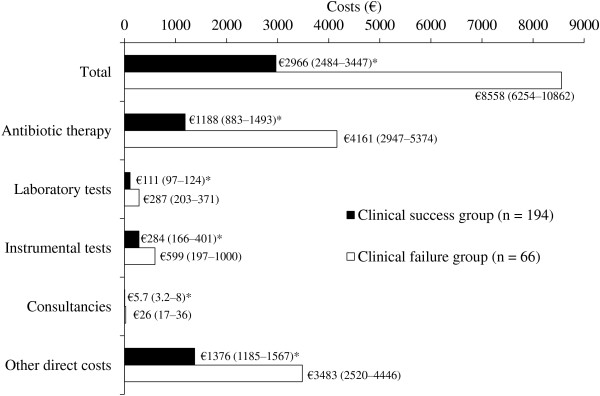
**Total hospitalization costs per patient, stratified by therapeutic outcome. Other direct costs category includes personnel, ordinary maintenance and hotel costs.** *p < 0.05 vs. clinical failure group.

Patients who failed antibiotic therapy received an average of 8.2 additional days of antibiotic therapy and spent 11 more days in hospital compared with patients who responded to first-line therapy (both p < 0.05 vs. clinical success group). Furthermore, they incurred €5592 in additional hospitalization costs (2.88 times the cost associated with clinical success) with 53% (€2973) of the additional costs attributable to antibiotic therapy (Figure 3). All of the other contributors to hospitalization costs were significantly higher in the clinical failure group (Figure 3).

**Figure 4:**
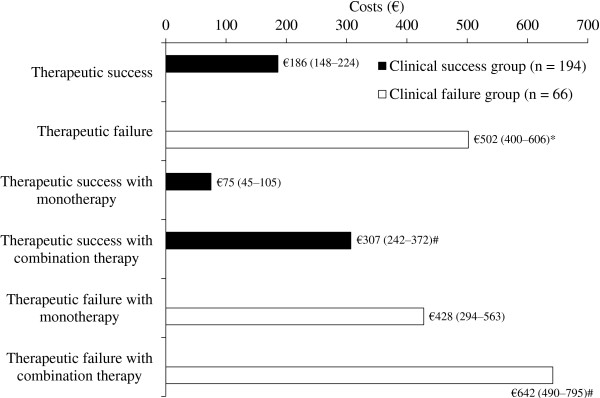
**Hospitalization costs per day of antibiotic therapy in patients stratified by therapeutic outcome and antibiotic regimens*.*** *p < 0.05 vs. clinical failure group; #p < 0.05 vs. antibiotic monotherapy group.

A significant cost difference was still found when average costs per hospital-day were computed, amounting to €386 (95% CI 350–421) for patients experiencing clinical success, and €476 (95% CI 414–539) for patients who failed first-line therapy. Antibiotic cost by itself still was a great contributor to total per day inpatient charges, in both success and failure groups (40% and 48.5%, respectively), being significantly higher in patients who failed starting therapy (€249 vs. €153).Due to the high contribution of antibiotic therapy to hospitalization costs, daily charges limited to antibiotic therapy course duration have been estimated (Figure 4), and were significantly higher for patients who clinically failed, as compared to those who succeeded (€502 vs. €186). This significant extra cost per antibiotic day in clinical failure cases was confirmed for both single and multiple drug antibiotic regimens (Figure 4).

## Discussion

To our knowledge, this is the first multicenter study investigating the economic outcome of hospitalized cIAIs in Italy. This study clearly shows that starting empirical antibiotic therapy has a large impact on the cost of care of community-acquired cIAIs. In this large sample of hospitalized adult patients with community-acquired cIAIs, clinical failure was the strongest independent predictor of increases in hospitalization costs. Compared with patients who are treated successfully, patients who failed therapy received antibiotic therapy for more than one additional week, spent 11 more days in hospital, and incurred a mean €5600 more in hospital charges. Antibiotic therapy was the leading contributor to inpatient charges, and multiple drug regimens was an independent predictor of increases in costs.

Various European and US studies have investigated the clinical outcomes associated with the treatment of community-acquired cIAIs and have shown a clinical failure rate of 17%–35% [2-5], which is consistent with the 25% failure rate observed in our study. However, very few studies have addressed the issue of the economic burden of cIAIs.

Early European series have shown that hospitalization costs are 1.2–1.5 times higher in patients who have failed treatment compared with patients who were treated successfully [2,6]. The present study confirms and substantiates these findings, demonstrating that the costs associated with failing first-line antibiotic therapy is associated with a 2.8-fold increase in hospitalization costs compared with patients who have had clinical success. Importantly, clinical failure was the strongest independent contributor to inpatient hospitalization charges, leading to an increase in costs of 87% after adjusting for comorbidities, therapeutic failure risk factors, type of primary surgical procedure and unscheduled additional surgeries.

The cost of antibiotic therapy explained over 50% of total hospitalization charges, confirming previous studies that have shown that antibiotics contribute 70% of extra costs associated with cIAIs [6]. This large proportion of clinical failure costs deriving from antibiotic therapy most probably arises from the overlap existing between the failure of antibiotic therapy and clinical failure. Although clinical failure, a widely employed measure of drug effectiveness [2-4,6,7], is a composite of three different outcomes (antibiotic therapy switch, re-operation or death), in most instances it is driven by failure of first-line antibiotic therapy. In our study virtually all patients who clinically failed required second-line antibiotic therapy, while re-operation or death involved only a few patients (17.7% and 9.1%, respectively). This is consistent with the results of previous studies which have shown that the majority of costs associated with clinical failure are due to antibiotic therapy [2,7].

In most cases, clinical failure with antibiotic therapy is driven by unsuitable drug choice [3,4,6]. In the present study, although only “presumed” basing on drug spectrum of coverage adequacy [1], appropriate antibiotic therapy was associated with a 78% chance of clinical success, compared with a 34% chance of clinical success associated with inappropriate therapy. Therefore, the role of antibiotic failure and inappropriateness of drug choice having a large influence on the occurrence of clinical failure could be inferred, as previously demonstrated [3,7,10].

As expected, the appropriateness of empiric antibiotic therapy was more frequently reached with wide spectrum combination therapy. We found that multiple-drug empiric regimens were appropriate in 97% of cases compared with roughly 65% of single drug regimens. Moreover, patients who achieved clinical success were more likely to have received antibiotic combination therapy than those patients who failed antibiotic therapy, confirming previous findings [7]. On the other hand, the costs per day of antibiotic combination regimens were significantly higher than the costs of antibiotic monotherapy, regardless of therapeutic outcome. Importantly, combination therapy was a strong independent predictor of increases in inpatient charges, causing approximately a 50% increase of mean hospitalization costs. Thus, the benefit/cost ratio underpinning the correct management of community-acquired cIAIs seems to be difficult to balance.

Multiple antibiotic regimens aim to expand antimicrobial spectrum and to overcome increased bacterial resistance in community-acquired cIAIs [13,14]. Recently, newly introduced wide spectrum agents, such as ertapenem and tygecicline, have been recommended [8] for use as first-line empiric antibiotic monotherapy regimens in stable, noncritically ill cIAIs patients with extended-spectrum beta-lactamase producing pathogens risk factors, factors that are becoming more frequently involved in community-acquired cIAIs [13,14]. Interestingly, these were also among the most frequent second-line choices in our failing patients, underlying the cost/benefit advantage of such recommendation.

As previously reported [2,6,7], patients who were less healthy due to an increased age, comorbidities or those with known treatment failure risk factors, were significantly more likely to fail antibiotic therapy. These same features independently increased hospitalization costs. Therefore, illness severity must be strongly considered when choosing starting empirical antibiotic therapy, due to its influence on clinical and economic outcomes of patients with cIAIs.

The low rate of intra-operative microbiology tests performed in the present study is worrisome. As choosing antibiotics for the treatment of cIAIs is an empiric decision, local epidemiology knowledge is of outmost importance. By increasing the chance of appropriate treatment [1], it could improve outcome and decrease resource utilization in patients subsequently hospitalized in the same institution for the same condition. Thus, we recommend that the consistent taking of swab samples by Italian surgeons is implemented.

As with any retrospective analysis, this study has several limitations. Due to complexities associated with the collection of data, summary measures of illness and comorbidities severity, potentially associated with clinical failure, longer length of hospital stay, and higher inpatient costs were not covered and could not be used in the multivariate model. We were also unable to assess the appropriateness of antibiotic therapy in light of culture results and patient clinical risk profile [1,9] and, therefore, the clinical failure variable, rather than antibiotic appropriateness, was used in the multivariable analysis of independent cost predictors. Finally, being a multicenter study, dissimilarity in standard of care among participating sites cannot be excluded.

Despite these limitations, for the first time we assessed patterns of starting antibiotic therapy, resource utilization and actual costs of caring for inpatients with community-acquired cIAIs in Italian hospitals. The results of this study suggest that hospitals need to be aware of the clinical and economic consequences of antibiotic therapy and to reduce overall resource use and costs by improving the rate of success with appropriate initial empiric therapy. Considering the prospective reimbursement system of the Italian NHS, there may be a relevant cost saving at the same reimbursement rate for hospitals, by reducing antibiotic costs of cIAIs. Mandatory peritoneal swab sampling, allowing for local epidemiology driven empiric antibiotic therapy, should be strongly encouraged for each cIAIs patient.

## Abbreviations

CI: Confidence interval; cIAIs: Complicated intra-abdominal infections; GI: Gastrointestinal; IAIs: Intra-abdominal infections; ICU: Intensive care unit; WBC: White blood cell.

## Competing interests

The authors LD, FB, EC, FR and CA declare that they have no competing interests. SC has received funds from Pfizer. FS has received research and educational grants from Abbott, Bayer, Biogen Idec, Biomarine, BMS, Boehringer-Ingelheim, Celgene, Daiichi Sankyo, Eli Lilly, Genzyme, GlaxoSmithKline, Janssen Cilag, Johnson & Johnson, Medtronic, MSD, Novartis, Novo Nordisk, Obi, Pfizer, Roche, Sanofi Pasteur Servier, Sigma Tau, Stroder, Teva. AN has received funds to support the research and donations from Pfizer, MSD and honorary from Astra Zeneca, MSD, Pfizer, Valeas and Zambon.

## Authors’ contributions

LD and CA carried out acquisition and interpretation of data, and drafted the manuscript; FB, SC, EC, AN, FR, and FS provided to conception and design of the study, and to manuscript revision; FS and CA performed the statistical analysis. All authors read and approved the final manuscript.
